# Effect of doxycycline and meloxicam on cytokines, brain-derived neurotrophic factor, matrix metalloproteinase-3, tissue inhibitor of metalloproteinase-3 and cyclooxygenase-2 in brain

**DOI:** 10.22038/ijbms.2020.45193.10527

**Published:** 2020-10

**Authors:** Ayse Er, Devran Coskun, Emre Bahcivan, Burak Dik

**Affiliations:** 1 Department of Pharmacology and Toxicology, Faculty of Veterinary Medicine, Selcuk University, Konya, Turkey; 2 Department of Pharmacology and Toxicology, Faculty of Veterinary Medicine, Siirt University, Siirt, Turkey; 3 Department of Pharmacology and Toxicology, Faculty of Veterinary Medicine, Kafkas University, Kars, Turkey

**Keywords:** Brain, Doxycycline, Inflammation, Meloxicam, Neuroprotective

## Abstract

**Objective(s):**

Prevention of inflammation in early stages will be useful in maintaining vitality of the organism. The objective of this study was to evaluate the effects of doxycycline (DOX) or meloxicam (MLX) monotherapy and combination therapy on the levels of inflammatory mediators in the brain tissues of rats with *Escherichia coli *lipopolysaccharide (LPS)-induced brain inflammation.

**Materials and Methods:**

Seventy-eight rats were divided into the following groups: control (n=6), LPS (0.5 μg/10 μl intracranial) (n=18), LPS (0.5 μg/10 μl intracranial)+DOX (40 mg/kg intraperitoneal) (n=18), LPS (0.5 μg/10 μl intracranial)+MLX (2 mg/kg intraperitoneal) (n=18) and LPS (0.5 μg/10 μl intracranial)+DOX (40 mg/kg intraperitoneal)+MLX (2 mg/kg intraperitoneal) (n=18) groups. Brain tissues were harvested from all rats in the control group and from six rats each in the four experimental groups at 1, 3 and 6 hr under anaesthesia. The levels of tumor necrosis factor α (TNFα), interleukin 4 (IL-4), IL-6, IL-10, IL-17, brain-derived neurotrophic factor (BDNF), matrix metalloproteinase 3 (MMP-3), tissue inhibitor of metalloproteinase 3 (TIMP-3) and cyclooxygenase 2 (COX-2) in the brain tissues were measured using ELISA kits with ELISA device.

**Results:**

LPS administration increased proinflammatory cytokines (TNF, IL-6, IL-17), and MMP-3 levels and decreased anti-inflammatory cytokines (IL-10, IL-4), and BDNF levels. The lowest TNFα levels were detected in the LPS+MLX group (*P<*0.05). All the drug treatment groups showed decreased IL-17 and COX-2 levels compared to the LPS groups.

**Conclusion:**

DOX or MLX monotherapy exerts neuroprotective effects against brain inflammation by decreasing proinflammatory cytokine levels and by increasing anti-inflammatory cytokines levels.

## Introduction

Neuroinflammation is an important factor in the pathogenesis and progression of neurodegenerative diseases. Patients’ brains with neurodegenerative diseases showed microglia activation, marked astrocytosis and increased proinflammatory cytokine levels (1). 

Lipopolysaccharide (LPS), an immunogenic component of gram-negative bacteria, is widely used to develop experimental models of neuroinflammation (1). LPS administration affects the levels of various inflammatory markers, including inflammatory cytokines, brain-derived neurotropic factor (BDNF), matrix metalloproteinase (MMP), tissue inhibitor of metalloproteinases (TIMP) and cyclooxygenase (COX) (2-4). Cytokines play important roles in inflammation and are divided into two groups as proinflammatory [Tumor necrosis factor (TNF) α, interleukin (IL)-6, IL-17, etc.] and anti-inflammatory (IL-4, IL-10, etc.) cytokines (5, 6). Increased TNF-α, IL-10 and IL-6 levels have been detected in the brain and cerebrospinal fluid of patients with acute infections of nervous system or in LPS- or *Streptococcus pneumonia*-infected models (7-10). COX-2 plays a central role in inflammation (11) and is overexpressed in neurodegenerative disorders such as Parkinson’s disease (12). Moreover, LPS administration increases COX activity (13). BDNF, a member of neurotrophin family, promotes the viability and differentiation of neurons (14), and BDNF level decreases during inflammation and in LPS-administrated rats (15). MMPs are proteolytic enzymes secreted in inactive form. These inactivate enzymes are activated during neuroinflammation by the action of free radicals, cytokines and other enzymes and are rapidly inactivated by TIMPs (16).

Tetracycline antibiotics exert bacteriostatic effect by inhibiting bacterial protein synthesis (17); moreover, they exert anti-inflammatory and neuroprotective effects (18). Doxycycline (DOX), a semi-synthetic tetracycline antibiotic, was administered intravenously and showed high lipophilicity and long half-life (18). Besides, DOX increased anti-inflammatory cytokine levels and decreased proinflammatory cytokine levels in an *in vitro* study (19). Meloxicam (MLX), a nonsteroidal anti-inflammatory drug, is often used for treating infections because of its potent analgesic, antipyretic and anti-inflammatory effects (20). Because MLX exerts a more portent effect on COX-2 than on COX-1, it is more effective for treating infections (21). Moreover, MLX exerts neuroprotective effects because of its antioxidant and anti-cytokine activity (21, 22).

Anti-inflammatory effects of MLX and DOX were associated with reduction of the proinflammatory cytokines and inhibition of the MMP in the brain and the experimental autoimmune neuritis (21, 23). DOX and MLX are two drugs that do not interact with each other and are used together (24). Because both MLX and DOX exert anti-inflammatory effects in nervous system, it was hypothesised that these drugs strongly inhibit the inflammatory mediators of brain inflammation. Therefore, the present study determined the effects of DOX (40 mg/kg intraperitoneal) (23) or MLX (2 mg/kg intraperitoneal) (25) monotherapy and combination therapy on the levels of TNF-α, IL-4, IL-6, IL-10, IL-17, BDNF, MMP-3, TIMP-3 and COX-2 in the brain tissues of rats with brain inflammation induced by intracranial LPS administration.

## Materials and Methods

***Animal and study design***

The study procedure was approved by the Ethics Committee of Experimental Medical Practice and Research Center of Animal Experiments of Selcuk University, Konya, Turkey (2016-38). This study included 78 male Wistar rats that were divided into the following groups: control (n=6), LPS (0.5 µg/10 µl intracranial) (n=18), LPS (0.5 µg/10 µl intracranial)+DOX (40 mg/kg IP, Doksimis 100 ml, Mistav) (n=18), LPS (0.5 µg/10 µl intracranial)+MLX (2 mg/kg IP, Maxicam X4, Sanovel) (n=18) and LPS (0.5 µg/10 µl intracranial) + DOX (40 mg/kg IP)+MLX (2 mg/kg IP) (n=18) groups. LPS, DOX and MLX were diluted by physiological saline solution. Brain tissues were harvested from all the six rats in the control group and from six rats each in the four experimental groups at 1, 3 and 6 hr. Acute phase response against acute infections develops especially within 6 hr in the body. Hence, we chose first 6 hr for determination of cytokine networks and other inflammatory mediators (26, 27). The rats were anaesthetised (70 mg/kg, thiopental, IP) and placed in a stereotaxic apparatus for intracranial LPS administration according to coordinates (0.8-mm posterior to the bregma and 1.5-mm lateral to the sagittal suture, 2.0 mm ventral to the skull surface) by using a Hamilton microsyringe. Next, the rats were intraperitoneally injected with the indicated drugs. The brain tissues were harvested by euthanising the anaesthetised rats through cervical dislocation and were stored at -80 ^°^C until further analyses.

***ELISA measurement***

The levels of TNFα, (Rat TNF-α ELISA kit, Catalog No: E-EL-R0019, Elabscience Biotechnology Co. Ltd., USA), IL-4 (Rat IL-4 ELISA kit, Catalog No: E-EL-R0014, Elabscience Biotechnology Co. Ltd., USA), IL-6 (Rat IL-6 ELISA kit, Catalog No: E-EL-R0015, Elabscience Biotechnology Co. Ltd., USA), IL-10 (Rat IL-10 ELISA kit, Catalog No: E-EL-R0016, Elabscience Biotechnology Co. Ltd., USA), IL-17 (Rat Il-17 ELISA kit, Catalog No: E-EL-R0566, Elabscience Biotechnology Co. Ltd., USA), BDNF (Rat BDNF ELISA kit, Catalog No: E-EL-R1235, Elabscience Biotechnology Co. Ltd., USA), COX-2 (Rat COX-2 ELISA kit, Catalog No: E-EL-R0792, Elabscience Biotechnology Co. Ltd., USA), MMP-3 (Rat MMP-3 ELISA kit, Catalog No: E-EL-R0619, Elabscience Biotechnology Co. Ltd., USA) and TIMP-3 (Rat TIMP-3 ELISA kit, Catalog No: E-EL-R0986, Elabscience Biotechnology Co. Ltd., USA) in homogenised brain tissue samples (0.5 g) were determined using commercial ELISA kits and an ELISA reader (MWGt Lambda Scan 200, USA) following manufacturer’s instructions.

***Statistical analysis***

Data are presented mean±SE and were evaluated using ANOVA and Duncan’s *post-hoc *test. All statistical analyses were performed using SPSS version 22.0, and *P*<0.05 was considered to be statistically significant.

## Results

The effects of DOX or MLX monotherapy and combination therapy on the levels of TNFα, IL-6, IL-17, IL-10, IL-4, COX-2, BDNF, MMP-3 and TIMP-3 in the brain tissues of the rats with LPS-induced brain inflammation are shown in Figure 1-9, respectively.

***DOX and MLX effect on TNF-α level in rat brain***

**Figure 1:**
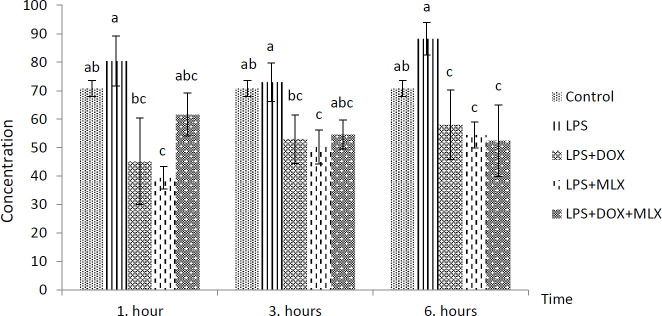
Effect of doxycycline and meloxicam administrations on brain tissue level of TNF-α (pg/ml/0.5 g tissue) in intracranial LPS-induced brain inflammation (mean±SE)

^a, b, c ^The different letters are statistically significant (*P<*0.05)

Increased TNF-α levels were determined in LPS group, but it was not statistically significant from control group in all the sampling times. However, TNF-α levels in the LPS+DOX and LPS+MLX groups were determined lower compared to the LPS group at all the sampling times (*P*<0.05) but only decreased at 6 hr in the rats of the LPS+DOX+MLX group (*P*<0.05) (Figure 1). 

***DOX and MLX effect on IL-6 level in rat brain***

**Figure 2:**
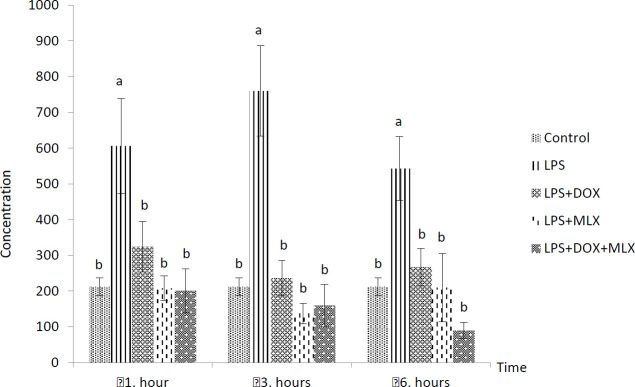
Effect of doxycycline and meloxicam administrations on brain tissue level of IL-6 (pg/ml/0.5 g tissue) in intracranial LPS-induced brain inflammation (mean±SE)

^a, b^ The different letters are statistically significant (*P<*0.05)

LPS administration significantly increased IL-6 levels at all the sampling times compared to the control group (*P*<0.05). This LPS-induced increase in IL-6 levels was inhibited in LPS+DOX, LPS+MLX and LPS+DOX+MLX groups at all the sampling times (*P*<0.05, Figure 2). 

***DOX and MLX effect on IL-17 level in rat brain***

**Figure 3:**
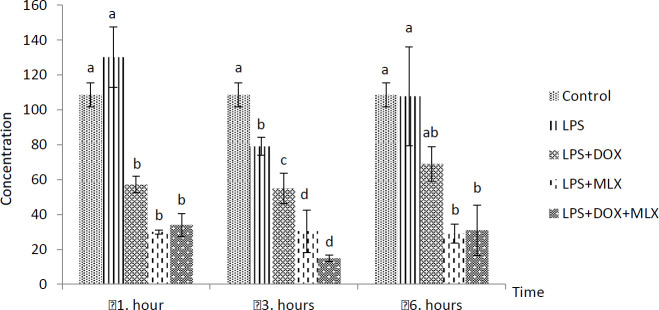
Effect of doxycycline and meloxicam administrations on brain tissue level of IL-17 (pg/m/0.5 g tissue) in intracranial LPS-induced brain inflammation (mean±SE)

^a, b, c, d ^The different letters are statistically significant (*P<*0.05)

LPS increased IL-17 level, but it was not statistically significant from control group at 1 hr. IL-17 level decreased in the LPS+DOX, LPS+MLX and LPS+DOX+MLX groups (*P*<0.05) compared to the LPS group (Figure 3). 

***DOX and MLX effect on IL-10 level in rat brain***

**Figure 4:**
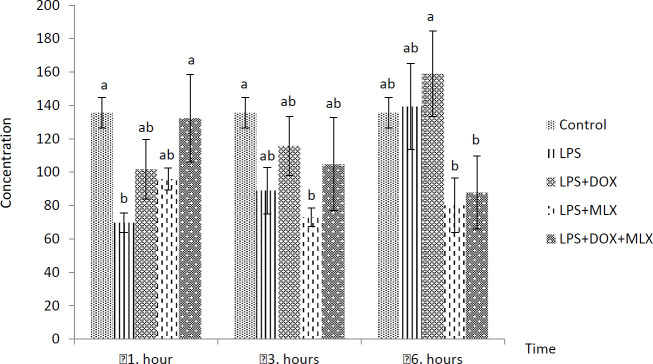
Effect of doxycycline and meloxicam administrations on brain tissue level of IL-10 (pg/ml/0.5 g tissue) in intracranial LPS-induced brain inflammation (mean±SE)

^a, b^ The different letters are statistically significant (*P<*0.05)

LPS administration resulted in a decrease in IL-10 levels for the first 3 hr, especially at 1 hr. Statistically significant changes were determined in the IL-10 levels in LPS+DOX, LPS+MLX and LPS+DOX+MLX compared to the LPS group at all the sampling times (Figure 4). 

***DOX and MLX effect on IL-4 level in rat brain***

**Figure 5:**
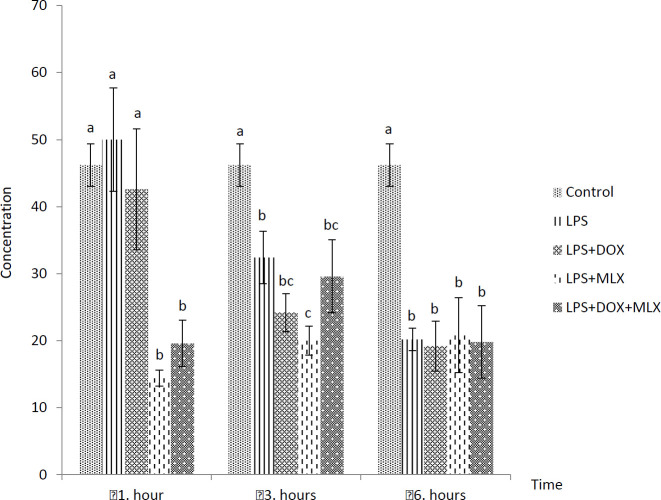
Effect of doxycycline and meloxicam administrations on brain tissue level of IL-4 (pg/ml/0.5 g tissue) in intracranial LPS-induced brain inflammation (mean±SE)

^a, b, c^ The different letters are statistically significant (*P<*0.05)

IL-4 is an anti-inflammatory cytokine, and LPS administration decreased its level. Drug treatments could not prevent this reduction at all the sampling times (Figure 5). 

***DOX and MLX effect on COX-2 level in rat brain***

**Figure 6:**
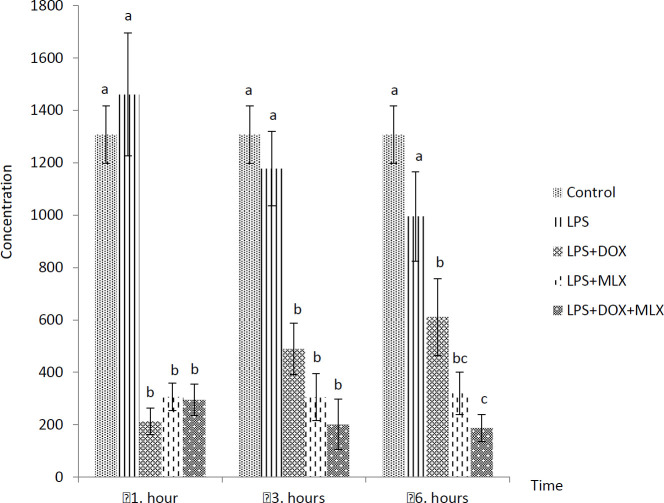
Effect of doxycycline and meloxicam administrations on brain tissue level of COX-2 (pg/ml/0.5 g tissue) in intracranial LPS-induced brain inflammation (mean±SE)

^a, b, c^ The different letters are statistically significant (*P<*0.05)

LPS increased COX-2 level at 1 hr, and the levels of COX-2 decreased at 3 and 6 hr, but these were not statistically significant from control group. COX-2 level decreased in the LPS+DOX, LPS+MLX and LPS+DOX+MLX groups (*P*<0.05) compared to the LPS group (Figure 6).

***DOX and MLX effect on BDNF level in rat brain***

**Figure 7:**
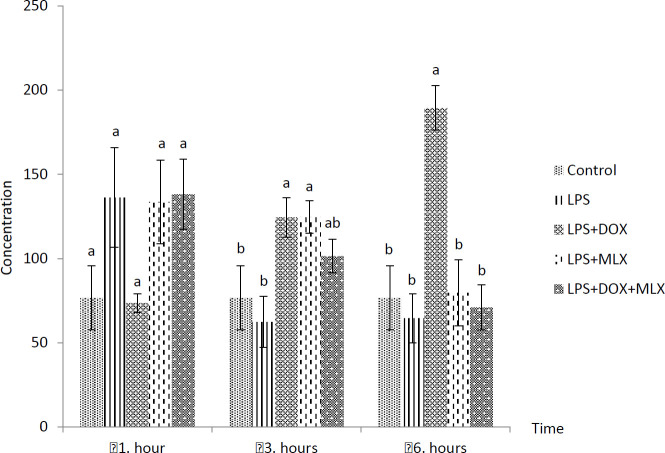
Effect of doxycycline and meloxicam administrations on brain tissue level of BDNF (pg/ml/0.5 g tissue) in intracranial LPS-induced brain inflammation (mean±SE)

^a, b^ The different letters are statistically significant (*P<*0.05)

LPS increased BDNF level at 1 hr and the levels of BDNF decreased at 3 and 6 hr, but these were not statistically significant from control group. The rats in the LPS+DOX group showed increased BDNF levels at 6 hr (*P*<0.05) compared to the rats in all the other groups. Moreover, BDNF levels increased in the LPS+DOX and LPS+MLX groups at 3 hr compared to the LPS group (*P*<0.05, Figure 7). 

***DOX and MLX effect on MMP-3 level in rat brain***

**Figure 8.**
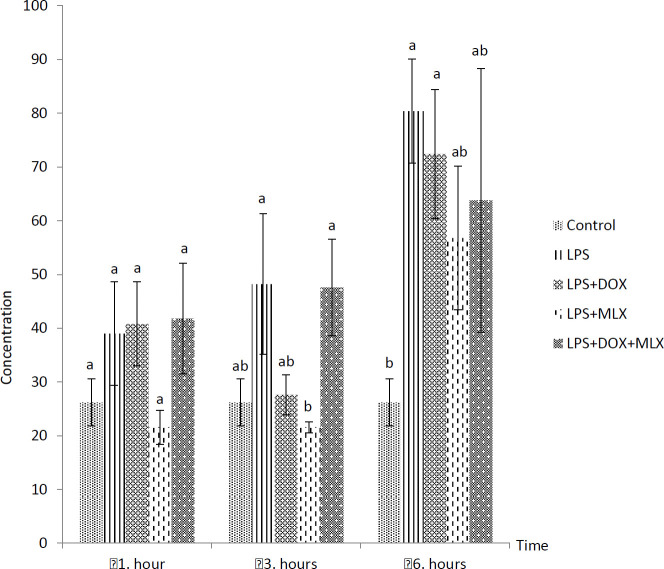
Effect of doxycycline and meloxicam administrations on brain tissue level of MMP-3 (ng/ml/0.5 g tissue) in intracranial LPS-induced brain inflammation (mean±SE)

^a, b^ The different letters are statistically significant (*P<*0.05)

MMP-3 levels increased in the LPS group after 6 hr (*P*<0.05) and decreased in the LPS+MLX group after 3 hr (*P*<0.05, Figure 8). 

***DOX and MLX effect on TIMP-3 level in rat brain***

**Figure 9:**
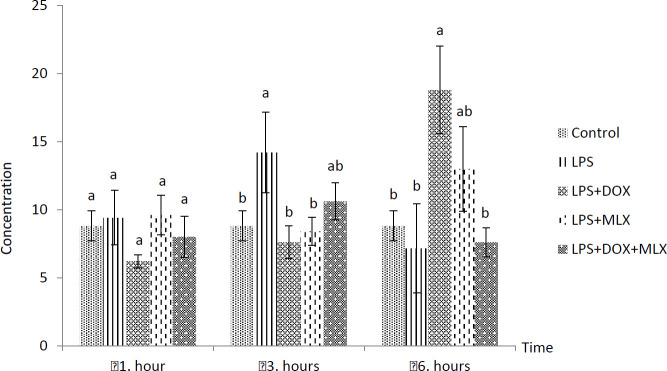
Effect of doxycycline and meloxicam administrations on brain tissue level of TIMP-3 (ng/ml/0.5 g tissue) in intracranial LPS-induced brain inflammation (mean±SE)

^a, b^ The different letters are statistically significant (*P<*0.05)

TIMP-3 levels increased in the LPS group after 3 hr (*P*<0.05) compared to control group. LPS+DOX and LPS+MLX caused decreased of TIMP-3 levels compared to LPS group after 3 hr (*P*<0.05) and LPS+DOX increased its level after 6 hr (*P*<0.05) (Figure 9).

LPS: Lipopolysaccharide, DOX: Doxycycline, MLX: Meloxicam, TNFα: Tumor necrosis factor α

LPS: Lipopolysaccharide, DOX: Doxycycline, MLX: Meloxicam, IL-6: Interleukin 6

LPS: Lipopolysaccharide, DOX: Doxycycline, MLX: Meloxicam, IL-17: Interleukin 17

LPS: Lipopolysaccharide, DOX: Doxycycline, MLX: Meloxicam, IL-10: Interleukin 10

LPS: Lipopolysaccharide, DOX: Doxycycline, MLX: Meloxicam, IL-4: Interleukin 4

LPS: Lipopolysaccharide, DOX: Doxycycline, MLX: Meloxicam, COX-2: Cyclooxygenase-2

LPS: Lipopolysaccharide, DOX: Doxycycline, MLX: Meloxicam, BDNF: Brain-derived neurotrophic factor

LPS: Lipopolysaccharide, DOX: Doxycycline, MLX: Meloxicam, MMP-3: Matrix metalloproteinase 3

LPS: Lipopolysaccharide, DOX: Doxycycline, MLX: Meloxicam, TIMP-3: Tissue inhibitor of metalloproteinase 3

## Discussion

LPS, a component of the cell wall of gram-negative bacteria, is a potent inducer of inflammation and exerts different effects on immune system cells (28). DOX and MLX are used in the human and veterinary medicine as antibacterial and anti-inflammatory drugs, respectively (20, 29). Although both the drugs have potent neuroprotective properties (18, 22), the neuroprotective effects of the combination of these drugs have not been investigated.

We use a positive control group in our studies with one sampling time. However, since there is not a single sampling time in this study (three sampling time), a positive control group should be formed at each sampling time. As a result, 18 animals would be used for 3 sampling times. For a total of 96 animals, the cost would double for each parameter. There is not enough budget in our projects, we could not add positive group. For this reason, as in our previous studies, due to the sterile study, the negative effects that may be related to the application were eliminated in this study. Hence, we used only one control group. In the present study, intracranial LPS administration increased the levels of TNF-α, IL-6 and IL-17, which are the main proinflammatory cytokines. In experimental studies, LPS is frequently used to develop models of brain inflammation (30). Intracranial LPS administration induces the secretion of inflammatory mediators from glial cells (28). Therefore, several studies have investigated the inflammatory mediators that play important roles in brain inflammation (30). Intracerebroventricular LPS administration increases IL-6 and TNFα levels in the brain tissue or cerebrospinal fluid (3, 31). Moreover, high IL-17 levels have been detected in the central nervous system during inflammation (5). Inhibition of proinflammatory cytokines during inflammation may help in alleviating pathological conditions (5). In the present study, DOX or MLX single drug administration (monotherapy) and combination therapy inhibited the LPS-induced increase in the levels of proinflammatory cytokines (TNFα, IL-6 and IL-17). Previous studies have also reported that DOX (23, 32) and MLX (21) decrease the levels of proinflammatory cytokines (TNFα, IL-6 and IL-17). In the present study, statistically significant changes were observed in levels of anti-inflammatory cytokines IL-10 and IL-4 (*P*<0.05). Generally, all the drug treatments increased IL-10 levels during the experimental period; however, a statistically significant increase in IL-10 levels was only observed in the rats of the LPS+DOX+MLX group after 1 hr. However, LPS decreased IL-4 level after 3 and 6 hr (*P*<0.05). The treatment groups were similar to the LPS group, except the LPS+MLX group. IL-4, an anti-inflammatory cytokine, in the LPS+MLX group decreased further after 3 hr. Similarly, some studies have reported unchanged cerebral IL-10 and IL-4 levels in patients with cerebral infections (33) and decreased cortical IL-10 levels in subjects treated with LPS (3). Some studies have reported increased cortical IL-10 levels after minocycline administration (3) and unchanged *Chlamydia trachomatis*-induced IL-10 expression after DOX administration (32). The anti-inflammatory effects of DOX and MLX may be associated with their depressor effects on nuclear factor-kappa B (34, 35). Nuclear factor-kappa B, a transcriptional factor, plays the main role in the activation of inflammatory mediators, including cytokines and chemokines, in the cell nucleus (36). 

In the present study, COX-2 levels were lower in the rats in the LPS+DOX, LPS+MLX and LPS+DOX+MLX groups than in the LPS group, whereas the levels of COX-2 in the control and LPS groups were similar (*P*<0.05). On the contrary to our result, increased COX-2 expression in the brain has been reported after LPS administrations (37, 38). This discrepancy in results may be associated with differences (such as animal kind, doses of drugs and/or sampling time) in the experimental design of the present and the previous studies. Decreased COX-2 levels have been reported after MLX (39, 40) and DOX (41) treatments. Because activated nuclear factor-kappa B stimulates COX-2 expression (42), the depressor effects of DOX and MLX on COX-2 synthesis may be explained by their inhibitory effect on nuclear factor-kappa B.

The LPS+DOX group showed significantly increased BDNF levels after 6 hr compared to the other groups (*P*<0.05). However, BDNF levels in the rats of the LPS+DOX and LPS+MLX groups were higher than the LPS group after 3 hr (*P*<0.05). Studies have reported decreased BDNF mRNA expression after intracerebral LPS administration (43) and increased BDNF levels after DOX and minocycline treatments (3, 44). In addition, MLX increases BDNF protein expression (31). In contrast, one study reported that DOX and minocycline treatments had no effect on decreased BDNF levels (45). Therefore, it can be suggested that DOX and MLX exert irregular effects on BDNF levels based on study conditions.

Although MMP-3 levels were similar in all the groups after 1 hr following the treatments, these levels increased in the LPS group after 6 hr. Moreover, MMP-3 levels in the brains of the treatment groups were similar to those in the LPS group but decreased in the rats of the LPS+MLX group after 3 hr. TIMP-3 levels increased in the rats of the LPS and LPS+DOX groups after 3 and 6 hr, respectively. MMPs play a role in neuronal physiology, cell viability and inflammation (46). In the central nervous system, the levels of most MMPs are negligible or low (46). However, some kinds of MMPs increase in glioma, viral infection, neuroinflammation, multiple sclerosis, Alzheimer’s disease, brain trauma and HIV-related neurological diseases (46). Active MMPs are rapidly inactivated by TIMPs (16), and MMP inhibition may prevent the progression of neuroinflammatory diseases (47). In addition, cytokines (TNF-α and IL-6) released from glial cells in infected areas increase MMP-3 and TIMP-3 expression (46, 48). The effect of DOX on MMP levels is controversial. Although DOX suppresses MMP-3 overexpression (49) and exerts a neuroprotective effect by decreasing MMP-3 levels in LPS-exposed microglial BV-2 cells (50), Lazzarini *et al.* (51) reported that DOX did not affect MMP-3 expression. The unimpressive effects of DOX and MLX on MMP-3 and TIMP-3 levels may be related to the doses of these drugs in the present study. 

## Conclusion

Our results suggest that DOX and MLX exert neuroprotective effects by decreasing proinflammatory cytokine (TNF-α, IL-6 and IL-17) levels and COX-2 synthesis in brain inflammation. In addition, the DOX and MLX combination therapy does not exert higher beneficial effects than their monotherapy.
